# Efficacy and safety of upadacitinib in patients with active psoriatic arthritis and axial involvement: results from two phase 3 studies

**DOI:** 10.1186/s13075-023-03027-5

**Published:** 2023-04-10

**Authors:** Xenofon Baraliakos, Roberto Ranza, Andrew Östör, Francesco Ciccia, Laura C. Coates, Simona Rednic, Jessica A. Walsh, Kevin Douglas, Tianming Gao, Koji Kato, In-Ho Song, Fabiana Ganz, Atul Deodhar

**Affiliations:** 1 Rheumazentrum Ruhrgebiet, Ruhr-University Bochum, Claudiusstr. 45, 44649 Herne, Germany; 2 Serviço de Reumatología, Hospital de Clinicas, Universidade Federal de Uberlândia, Uberlândia, Minas Gerais Brazil; 3 Monash University, Cabrini Hospital & Emeritus Research, Melbourne & ANU, Canberra, Australia; 4 University of Campania Luigi Vanvitelli, Caserta, Italy; 5 Nuffield Department of Orthopaedics, Rheumatology and Musculoskeletal Sciences, University of Oxford, Oxford, UK; 6 Rheumatology Department, Iuliu Hatieganu University of Medicine and Pharmacy, Cluj-Napoca, Romania; 7 Salt Lake City Veterans Affairs Health, Salt Lake City, UT USA; 8 University of Utah Health, Salt Lake City, UT USA; 9 AbbVie Inc, North Chicago, IL USA; 10 AbbVie Inc, Baar, Switzerland; 11 Oregon Health & Science University, Portland, OR USA

**Keywords:** Adalimumab, Ankylosing Spondylitis Disease Activity Score (ASDAS), Axial involvement, Bath Ankylosing Spondylitis Disease Activity Index (BASDAI), Janus kinase (JAK) inhibitor, Psoriatic arthritis (PsA), Safety, Upadacitinib

## Abstract

**Background:**

The objective of this post-hoc analysis was to assess the efficacy and safety of upadacitinib in psoriatic arthritis (PsA) patients with axial involvement.

**Methods:**

Post-hoc analysis of SELECT-PsA 1 and SELECT-PsA 2 in patients randomized to upadacitinib 15 mg (UPA15), placebo (switched to UPA15 at week 24), or adalimumab 40 mg (ADA; SELECT-PsA 1 only). Axial involvement was determined by investigator judgement (yes or no; based on the totality of available clinical information, such as duration and characteristics of back pain, age of onset, and previous lab investigations and imaging, if available) alone, or investigator judgement and patient-reported outcome (PRO)-based criteria (Bath Ankylosing Spondylitis Disease Activity Index [BASDAI] ≥ 4 and BASDAI Q2 ≥ 4). Efficacy outcomes that describe axial disease activity, including BASDAI endpoints, such as change from baseline in the overall BASDAI score or proportion of patients achieving BASDAI50 (≥ 50% improvement from baseline), as well as Ankylosing Spondylitis Disease Activity Score (ASDAS) endpoints, such as mean change from baseline in overall ASDAS or proportion of patients achieving ASDAS inactive disease or low disease activity, were evaluated at weeks 12, 24, and 56, with nominal *P*-values shown. Treatment-emergent adverse events (TEAEs) are summarized through week 56.

**Results:**

30.9% of patients in SELECT-PsA 1 and 35.7% in SELECT-PsA 2 had axial involvement by investigator judgement alone; 22.6% (SELECT-PsA 1) and 28.6% (SELECT-PsA 2) had axial involvement by investigator judgement and PRO-based criteria. Greater proportions of patients achieved BASDAI50 with UPA15 versus placebo using either criterion, and versus ADA using investigator judgement alone, at week 24 in SELECT-PsA 1 (investigator alone: UPA15, 59.0%, placebo, 26.9%, *P* < 0.0001, ADA, 44.1%, *P* = 0.015; investigator and PRO-based: UPA15, 60.4%, placebo, 29.3%, *P* < 0.0001, ADA, 47.1%, *P* = 0.074), with comparable findings in SELECT-PsA 2. Similar results were observed with UPA15 for additional BASDAI and ASDAS endpoints at weeks 12 and 24, with improvements maintained at week 56. Rates of TEAEs were generally similar across sub-groups irrespective of axial involvement status.

**Conclusions:**

PsA patients with axial involvement determined by predefined criteria showed greater BASDAI and ASDAS responses with UPA15 versus placebo, and numerically similar/greater responses versus ADA. Safety results were generally comparable between patients with or without axial involvement.

**Trial registration:**

ClinicalTrials.gov: SELECT-PsA 1, NCT03104400; SELECT-PsA 2, NCT0310437.

**Supplementary Information:**

The online version contains supplementary material available at 10.1186/s13075-023-03027-5.

## Background

Currently, there are no commonly accepted criteria for identifying axial involvement in psoriatic arthritis (PsA), and prevalence rates range from 5 to 70% depending on the criteria used for diagnosis and disease duration [1–6]. Despite this lack of consensus, identifying and effectively treating PsA patients with axial involvement remains an important clinical topic. Registry data suggest that PsA patients with axial involvement had higher disease activity and greater impairment in quality of life compared to PsA patients without axial involvement [7]. Furthermore, axial involvement in PsA responds differently to certain treatments compared to peripheral PsA [1, 8]. Therefore, a better understanding of clinical responses in patients with PsA and axial involvement can help optimize treatment decisions.

The efficacy of upadacitinib, an oral Janus kinase (JAK) inhibitor, to treat adults with active PsA or axial spondyloarthritis (axSpA) has been established in phase 3 clinical trials, with a consistent long-term safety profile observed across rheumatoid arthritis, PsA, and axSpA [9–23]. Given the observed benefits of upadacitinib for the treatment of PsA, and the need to better understand clinical responses in PsA patients with axial involvement, the objective of this post-hoc analysis was to assess the efficacy and safety of upadacitinib in patients with active PsA and axial involvement from the phase 3 SELECT-PsA trials.

## Methods

Full methodological details for SELECT-PsA 1 (NCT03104400) and SELECT-PsA 2 (NCT03104374), including study dates and size, inclusion/exclusion criteria, randomization and blinding, and concomitant treatments have been published previously [20, 21].

### Patients and trial design

Adults (≥ 18 years) with a clinical diagnosis of active PsA, who also fulfilled the Classification Criteria for Psoriatic Arthritis (CASPAR), [24] and had an inadequate response or intolerance to ≥ 1 non-biologic disease-modifying antirheumatic drug (non-bDMARD) (SELECT-PsA 1) or ≥ 1 bDMARD (SELECT-PsA 2) were eligible for inclusion in these trials. Patients were randomized to receive blinded once daily oral upadacitinib 15 mg or upadacitinib 30 mg, placebo, or every other week subcutaneous adalimumab 40 mg (SELECT-PsA 1 only) for 24 weeks. At week 24, patients assigned to placebo at baseline were switched to blinded upadacitinib 15 mg or 30 mg. Blinding was maintained until all patients reached week 56. Open-label long-term extensions are currently ongoing for both trials (SELECT-PsA 1 up to 5 years; SELECT-PsA 2 up to 3 years).

Both studies were conducted in accordance with the International Council for Harmonisation of Technical Requirements for Pharmaceuticals for Human Use (ICH) guidelines, applicable regulations governing clinical trial conduct, and the Declaration of Helsinki 1964 and its later amendments. As per Good Clinical Practice (GCP), the trial protocols were approved by an independent ethics committee (IEC)/institutional review board (IRB). All patients provided written informed consent.

### Axial involvement

At baseline, axial involvement was determined by investigator judgement (yes or no) based on the totality of available clinical information, such as duration and characteristics of back pain, age of onset, and previous lab investigations and imaging, if available. For these studies, imaging was not required to confirm axial disease. In addition to investigator judgement, a second set of patient-reported outcome (PRO)-based criteria for active axial inflammatory involvement were applied: overall BASDAI score ≥ 4 and BASDAI question 2 (“*How would you describe the overall level of AS (ankylosing spondylitis) neck, back, or hip pain you have had?*”) score ≥ 4 at baseline [25].

### Outcomes

Efficacy outcomes that describe axial disease activity in the two PsA patient sub-groups with axial involvement (ie, axial involvement defined by investigator judgement alone or investigator judgement and PRO-based criteria) were evaluated at weeks 12, 24, and 56 for both studies. Efficacy endpoints included change from baseline in the overall BASDAI score, modified BASDAI score (excluding question 3 related to peripheral joint pain), and the Ankylosing Spondylitis Disease Activity Score (ASDAS [CRP], hereafter referred to as ASDAS). In addition, change from baseline in individual BASDAI components (questions 1 to 6) were evaluated. The proportion of patients achieving BASDAI50 (≥ 50% improvement from baseline in BASDAI), as well as ASDAS inactive disease (ID, defined as score < 1.3), ASDAS low disease activity (LDA, defined as score < 2.1), ASDAS major improvement (MI, defined as ≥ 2.0 decrease from baseline), and ASDAS clinically important improvement (CII, defined as ≥ 1.1 decrease from baseline) were also assessed.

For this post-analysis, safety data from both SELECT-PsA trials were summarized for all patients who received ≥ 1 dose of study drug through week 56. Treatment-emergent adverse events (TEAEs) were coded per the Medical Dictionary for Regulatory Activities (MedDRA; v 22.0) and graded using the National Cancer Institute Common Terminology Criteria for Adverse Events (CTCAE; v 5.0). Deaths and cardiovascular events were adjudicated by a blinded, independent, external committee using definitions that were pre-specified. Reported gastrointestinal (GI) perforations were adjudicated by a blinded committee of sponsor-employed experts.

### Statistical analysis

For this post-hoc analysis, efficacy endpoints were summarized for randomized patients who received at least one dose of trial drug. Data from patients treated with placebo, upadacitinib 15 mg, and adalimumab 40 mg (SELECT-PsA 1 only) at weeks 12 and 24 are presented, as well as placebo switched to upadacitinib 15 mg, continuous upadacitinib 15 mg, and adalimumab 40 mg (SELECT-PsA 1 only) at week 56. As upadacitinib 15 mg is the approved dose for patients with active PsA, [26] baseline demographics and efficacy data from patients treated with continuous upadacitinib 30 mg are not shown.

Continuous efficacy endpoints (change from baseline in overall BASDAI, modified BASDAI, individual BASDAI components, and ASDAS) were assessed using mixed-effect model for repeated measures (MMRM) analysis with unstructured variance–covariance matrix, including treatment, visit, treatment-by-visit interaction, and the stratification factor current DMARD use (yes/no) as fixed factors, and the continuous fixed covariate of baseline measurement. Least squares means with 95% confidence intervals (CIs) are provided for the continuous efficacy endpoints. The MMRM analysis for week 24 excluded data collected after premature discontinuation of the study drug. Binary efficacy endpoints (proportions of patients achieving BASDAI50, ASDAS ID, ASDAS LDA, ASDAS MI, and ASDAS CII) were analyzed using Cochran-Mantel–Haenszel tests adjusted for the main stratification factor of current DMARD use (yes/no). Binary efficacy endpoints are summarized as response rates with 95% CIs. Non-responder imputation (NRI) was used for missing data handling of binary endpoints. Differences between upadacitinib 15 mg and placebo at weeks 12 and 24 (SELECT-PsA 1 and SELECT-PsA 2), as well as upadacitinib 15 mg and adalimumab at weeks 12, 24, and 56 (SELECT-PsA 1 only), were based on nominal *P* values and were not multiplicity controlled. All data were analyzed using SAS version 9.4 (Cary, NC, USA).

Exposure-adjusted event rates (EAERs; events per 100 patient-years) of TEAEs (with 95% CIs) are summarized for patients who received ≥ 1 dose of study drug through week 56 in SELECT-PsA 1 and SELECT-PsA 2. Data for upadacitinib 15 mg (including patients who were assigned to placebo at baseline and switched to upadacitinib 15 mg treatment at week 24) and adalimumab (SELECT-PsA 1 only) are shown across all sub-groups (ie, without axial involvement, with axial involvement defined by investigator judgement alone, or with axial involvement defined by investigator judgement and PRO-based criteria).

## Results

### Patient sub-groups and baseline status

**Fig. 1:**
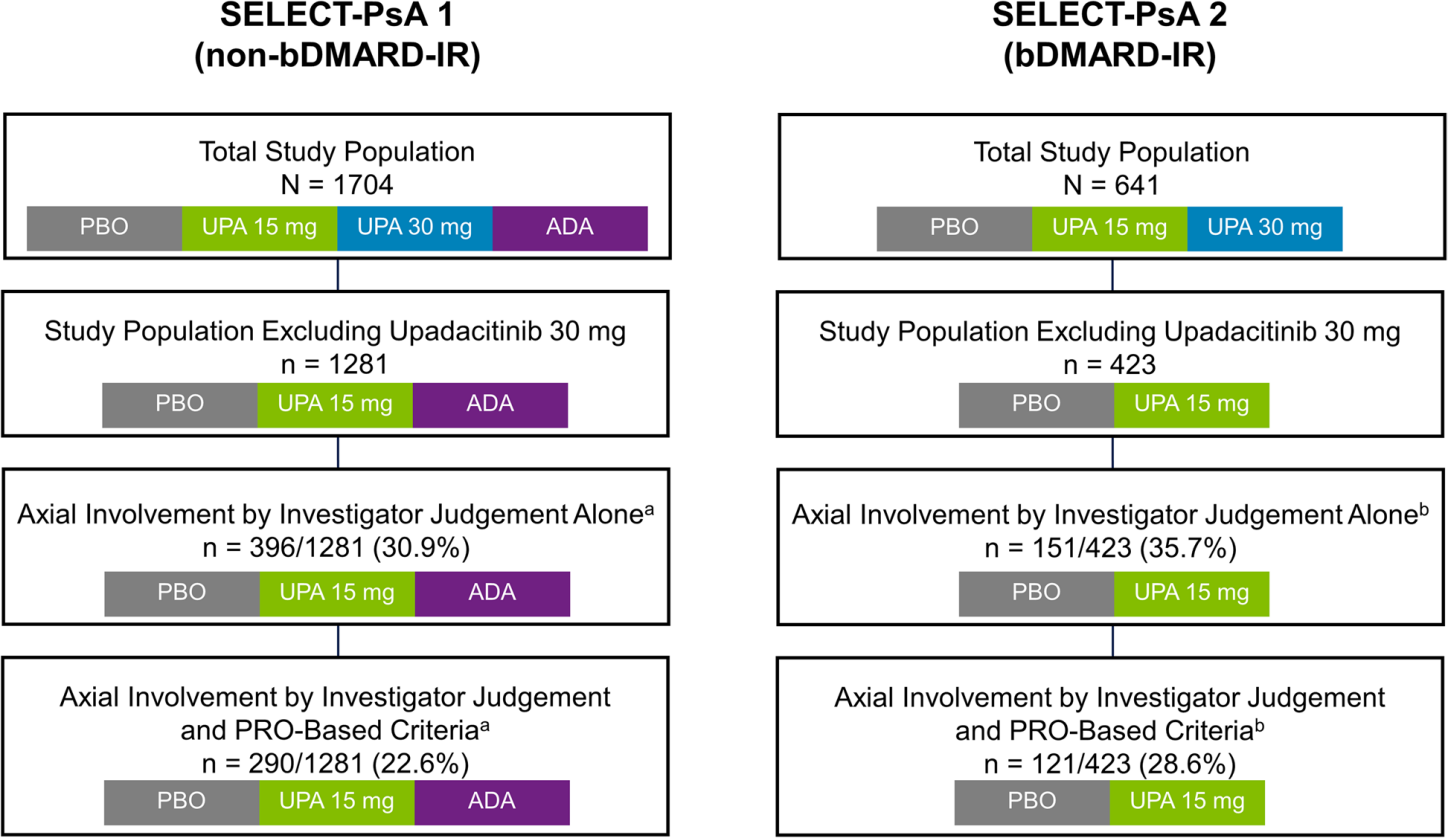
Proportion of PsA Patients With Axial Involvement Defined by Investigator Judgement Alone or Investigator Judgement and PRO-Based Criteria. ^a^Sub-groups shown include the following treatments from SELECT-PsA 1: placebo, adalimumab, and upadacitinib 15 mg; upadacitinib 30 mg is excluded. ^b^Sub-groups shown include the following treatments from SELECT-PsA 2: placebo and upadacitinib 15 mg; upadacitinib 30 mg is excluded. ADA, adalimumab; bDMARD, biologic disease-modifying antirheumatic drug; IR, inadequate response; PBO, placebo; PRO, patient-reported outcome; PsA, psoriatic arthritis; UPA, upadacitinib

Of the patient populations in each study (excluding continuous upadacitinib 30 mg), 30.9% (*n* = 396/1281) of PsA patients in SELECT-PsA 1 (non-bDMARD-IR [inadequate response]) and 35.7% (*n* = 151/423) of patients in SELECT-PsA 2 (bDMARD-IR) were defined as having axial involvement based on investigator judgement alone. Based on investigator judgement and PRO-based criteria, 22.6% (*n* = 290/1281) of PsA patients in SELECT-PsA 1 and 28.6% (*n* = 121/423) of patients in SELECT-PsA 2 were defined as having axial involvement (Fig. 1A, B). Mean age at baseline was slightly higher in the bDMARD-IR sub-groups regardless of axial involvement (range: 51.9 to 54.5 years old; non-bDMARD-IR range: 50.0 to 51.5 years old), over half of patients in each sub-group were female (range: 50.3 to 56.3%), and the majority of patients were White (range: 84.1 to 90.5%) (Table 1). At baseline, PsA patients with axial involvement based on either criterion showed marginally higher disease burden than patients without axial involvement, but other baseline characteristics were comparable across all groups. By definition, patients with axial involvement defined by both investigator judgement and PRO-based criteria had higher BASDAI scores at baseline than those defined by investigator judgement alone.

**Table 1:** Baseline Demographics and Disease Characteristics

**Parameter, mean (SD)**^c^	**SELECT-PsA 1 (non-bDMARD-IR)**^a^			**SELECT-PsA 2 (bDMARD-IR)**^b^		
	Without Axial Involvement (*n* = 885)	Axial Involvement by Investigator Alone (*n* = 396)	Axial Involvement by Investigator + PRO-Based (*n* = 290)	Without Axial Involvement (*n* = 272)	Axial Involvement by Investigator Alone (*n* = 151)	Axial Involvement by Investigator + PRO-Based (*n* = 121)
Age, years	51.5 (11.7)	50.1 (13.0)	50.0 (12.7)	54.5 (11.6)	51.9 (12.0)	52.2 (11.9)
Sex, n (%)						
Female	472 (53.3)	199 (50.3)	153 (52.8)	153 (56.3)	80 (53.0)	67 (55.4)
Male	413 (46.7)	197 (49.7)	137 (47.2)	119 (43.8)	71 (47.0)	54 (44.6)
Race, n (%)						
American Indian or Alaska Native	2 (0.2)	2 (0.5)	1 (0.3)	2 (0.7)	1 (0.7)	1 (0.8)
Asian	71 (8.0)	44 (11.1)	27 (9.3)	19 (7.0)	17 (11.3)	10 (8.3)
Black or African American	1 (0.1)	5 (1.3)	4 (1.4)	7 (2.6)	5 (3.3)	4 (3.3)
Multiple	9 (1.0)	6 (1.5)	6 (2.1)	0	1 (0.7)	1 (0.8)
Native Hawaiian or Other Pacific Islander	1 (0.1)	2 (0.5)	1 (0.3)	2 (0.7)	0	0
White	801 (90.5)	337 (85.1)	251 (86.6)	242 (89.0)	127 (84.1)	105 (86.8)
Ethnicity, n (%)						
Hispanic or Latino/a	146 (16.5)	44 (11.1)	37 (12.8)	52 (19.1)	37 (24.5)	30 (24.8)
Not Hispanic or Latino/a	739 (83.5)	352 (88.9)	253 (87.2)	220 (80.9)	114 (75.5)	91 (75.2)
BMI (kg/m^2^)	30.5 (7.0)	30.1 (6.5)	30.5 (6.4)	31.5 (7.1)	31.8 (8.1)	32.6 (8.4)
Duration of PsA diagnosis, years	6.0 (7.1)	6.3 (7.2)	6.9 (7.7)	10.5 (9.5)	9.9 (9.2)	10.4 (9.7)
Duration of PsA symptoms, years	8.9 (8.5)	9.9 (9.0)	10.0 (8.9)	13.8 (10.9)	12.7 (9.4)	13.0 (9.9)
Overall BASDAI	5.3 (2.2)	5.8 (2.0)	6.7 (1.5)	5.9 (2.2)	6.2 (2.0)	6.9 (1.4)
BASDAI Q2 (neck, back, or hip pain)^d^	4.6 (3.2)	5.8 (2.8)	7.1 (1.6)	5.5 (3.2)	6.4 (2.7)	7.4 (1.7)
BASDAI Q3 (pain in joints other than neck, back, or hip)^d^	5.9 (2.6)	6.1 (2.4)	6.9 (1.9)	6.2 (2.6)	6.3 (2.3)	6.9 (1.8)
ASDAS (CRP)	3.1 (1.0)	3.4 (1.0)	3.7 (0.8)	3.2 (1.1)	3.4 (1.0)	3.6 (0.8)
Patient’s assessment of pain^d^	6.0 (2.1)	6.2 (2.0)	6.7 (1.7)	6.4 (2.2)	6.6 (2.0)	6.9 (1.8)
Patient’s global assessment of disease activity^d^	6.4 (2.0)	6.4 (2.1)	7.0 (1.8)	6.7 (2.0)	7.0 (1.9)	7.2 (1.8)
Physician’s global assessment of disease activity^d^	6.5 (1.6)	6.7 (1.7)	6.9 (1.6)	6.4 (1.8)	6.7 (1.8)	7.0 (1.6)
TJC68	19.3 (13.6)	22.0 (15.6)	23.5 (16.1)	23.3 (16.7)	28.2 (18.4)	30.0 (18.8)
SJC66	11.1 (8.1)	12.2 (10.1)	12.8 (10.7)	11.0 (8.0)	12.9 (9.3)	12.7 (9.7)
Presence of dactylitis,^e^ n (%)	245 (27.7)	144 (36.4)	101 (34.8)	64 (23.5)	55 (36.4)	45 (37.2)
Presence of enthesitis,^f^ n (%)	665 (75.1)	320 (80.8)	236 (81.4)	218 (80.1)	128 (84.8)	108 (89.3)
BSA with psoriasis, n (%)						
< 3%	460 (52.0)	185 (46.7)	128 (44.1)	103 (37.9)	59 (39.1)	51 (42.1)
≥ 3%	425 (48.0)	211 (53.3)	162 (55.9)	169 (62.1)	92 (60.9)	70 (57.9)
hsCRP (mg/L)	10.5 (14.2)	12.6 (17.6)	13.2 (19.0)	11.0 (18.9)	10.4 (17.8)	11.6 (19.5)

*ASDAS* Ankylosing Spondylitis Disease Activity Score, *BASDAI* Bath Ankylosing Spondylitis Disease Activity Index, *bDMARD* Biologic disease-modifying antirheumatic drug, *BSA* Body surface area, *hsCRP* High-sensitivity CRP, *IR* Inadequate response, *PRO* Patient-reported outcome, *PsA* Psoriatic arthritis, *SJC66* swollen joint count 66, *TJC68* Tender joint count 68

^a^Sub-groups shown include the following treatments from SELECT-PsA 1: placebo, adalimumab, and upadacitinib 15 mg

^b^Sub-groups shown include the following treatments from SELECT-PsA 2: placebo and upadacitinib 15 mg

^c^Data presented as mean (SD) unless indicated

^d^Measured via numeric rating scale, 0 to 10

^e^As determined by Leeds Dactylitis Index > 0

^f^As determined by Total Enthesitis Count > 0

### Efficacy endpoints

**Fig. 2:**
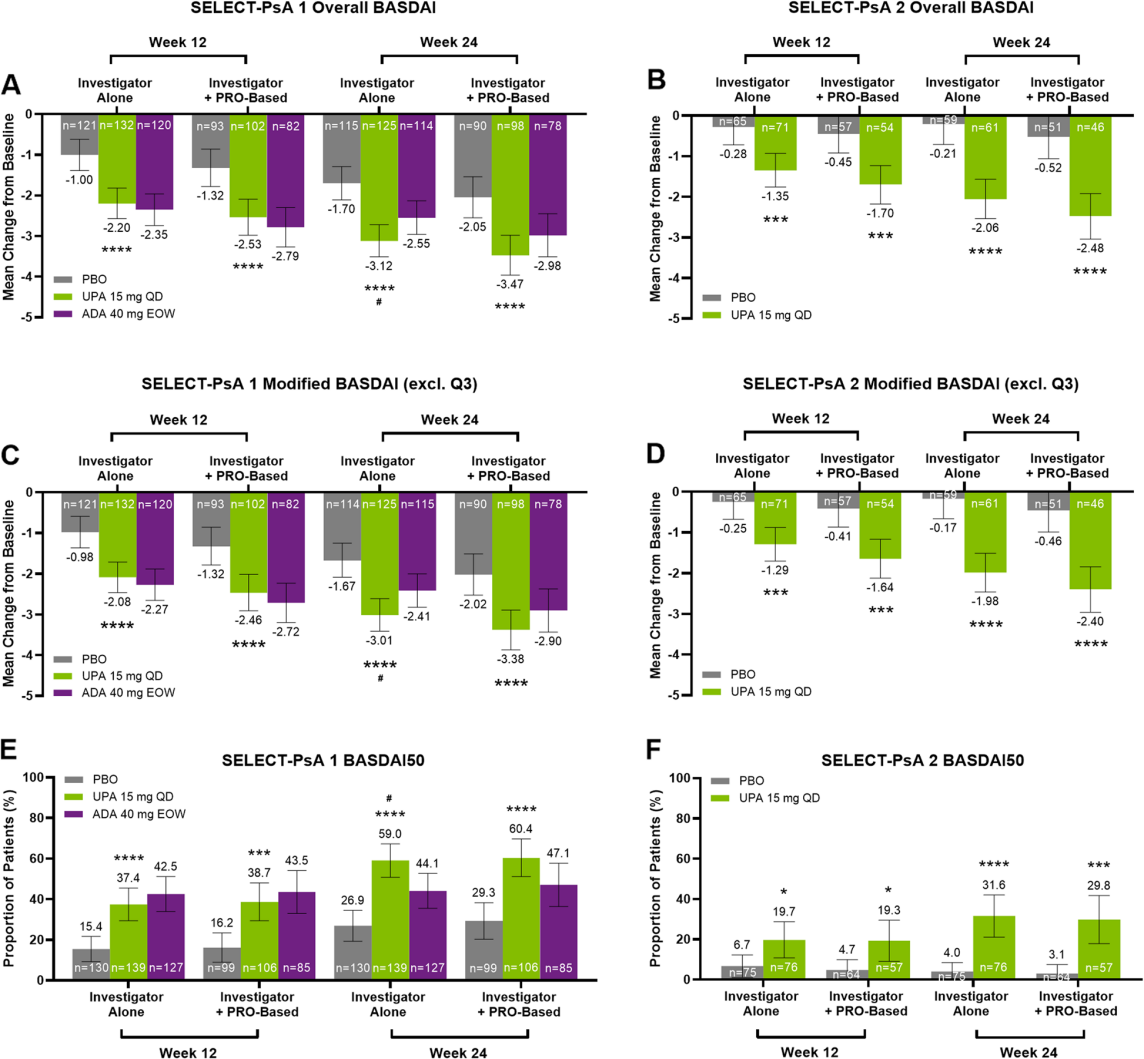
Mean Change From Baseline in Overall BASDAI and Modified BASDAI (Excluding Question 3) Scores, and Proportion of Patients Achieving BASDAI50, at Weeks 12 and 24 From SELECT-PsA 1 (non-bDMARD-IR) and SELECT-PsA 2 (bDMARD-IR). Mean change from baseline in the overall BASDAI score (**A**) or modified BASDAI (excluding question 3—How would you describe the overall level of pain/swelling in joints other than neck, back, or hips you have had?) score (**C**) at weeks 12 and 24 for PsA patients with axial involvement defined by investigator judgement alone or investigator judgement and PRO-based criteria treated with placebo, upadacitinib 15 mg QD, or adalimumab 40 mg EOW from SELECT-PsA 1. Mean change from baseline in overall BASDAI (**B**) or modified BASDAI (**D**) at weeks 12 and 24 for PsA patients with axial involvement defined by either criterion treated with placebo or upadacitinib 15 mg QD from SELECT-PsA 2. Proportions of PsA patients with axial involvement defined by investigator judgement alone or investigator judgement and PRO-based criteria that achieved BASDAI50 at weeks 12 and 24 treated with placebo, upadacitinib 15 mg QD, or adalimumab 40 mg EOW from SELECT-PsA 1 (**E**) or with placebo or upadacitinib 15 mg QD from SELECT-PsA 2 (**F**). Overall BASDAI and modified BASDAI were analyzed using mixed-effect model for repeated measures and are shown as least squares means with 95% CIs. BASDAI50 was analyzed using Cochran-Mantel–Haenszel tests with non-responder imputation and shown as response rates with 95% CIs. ****P < 0.0001, ***P < 0.001, *P < 0.05 upadacitinib 15 mg versus placebo; ^#^*P* < 0.05, upadacitinib 15 mg versus adalimumab; nominal *P* values are shown and were not multiplicity controlled. ADA, adalimumab; BASDAI, Bath Ankylosing Spondylitis Disease Activity Index; BASDAI50, ≥ 50% improvement from baseline in BASDAI; bDMARD, biologic disease-modifying antirheumatic drug; CI, confidence interval; EOW, every other week; IR, inadequate response; PBO, placebo; PRO, patient-reported outcome; PsA, psoriatic arthritis; QD, once daily; UPA, upadacitinib

Overall BASDAI score improvements were greater with upadacitinib 15 mg versus placebo at weeks 12 and 24 in both studies and according to both criteria used to define axial involvement (Fig. 2A, B), and versus adalimumab at week 24 in PsA patients with axial involvement defined by investigator judgement alone (Fig. 2A). Mean change from baseline in modified BASDAI (excluding question 3 related to peripheral joint pain) was greater with upadacitinib 15 mg versus placebo in PsA patients with axial involvement defined by both criteria at both time points and across both studies (Fig. 2C, D). Results for the individual BASDAI components (questions 1 to 6) were similar and consistent with that observed for overall BASDAI and modified BASDAI (Supplementary Fig. S1A-D, Supplementary Fig. S2A-D), with a greater response observed for BASDAI question 2 (related to back pain) at week 24 with upadacitinib 15 mg versus adalimumab based on investigator judgement alone (Supplementary Fig. 1C). A greater proportion of PsA patients achieved a BASDAI50 response with upadacitinib 15 mg versus placebo at weeks 12 and 24 in both studies regardless of the criteria used to define axial involvement (Fig. 2E, F), and versus adalimumab at week 24 based on investigator judgement alone (Fig. 2E). In general, clinical responses for overall BASDAI, modified BASDAI, and BASDAI50 were similar with upadacitinib 15 mg and adalimumab at week 12 but were numerically higher with upadacitinib 15 mg than adalimumab at week 24, irrespective of the criteria used to define axial involvement.

**Fig. 3:**
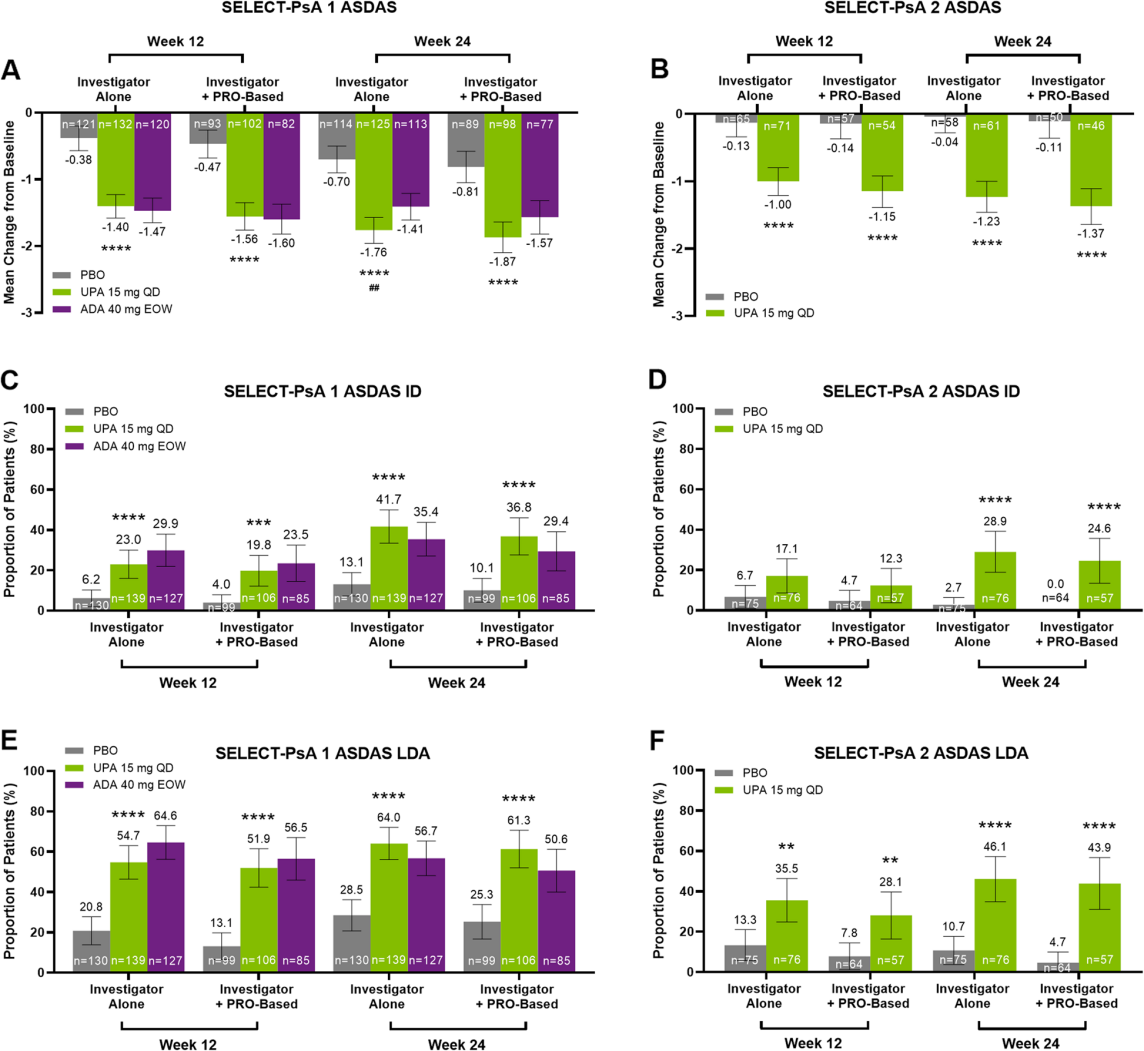
Mean Change From Baseline in ASDAS Scores, and Proportion of Patients Achieving ASDAS Inactive Disease (ID) and ASDAS Low Disease Activity (LDA), at Weeks 12 and 24 From SELECT-PsA 1 (non-bDMARD-IR) and SELECT-PsA 2 (bDMARD-IR). Mean change from baseline in ASDAS (CRP) scores at weeks 12 and 24 for PsA patients with axial involvement defined by investigator judgement alone or investigator judgement and PRO-based criteria treated with placebo, upadacitinib 15 mg QD, or adalimumab 40 mg EOW from SELECT-PsA 1 (**A**) or with placebo or upadacitinib 15 mg QD from SELECT-PsA 2 (**B**). Proportions of PsA patients with axial involvement defined by investigator judgement alone or investigator judgement and PRO-based criteria treated with placebo, upadacitinib 15 mg QD, or adalimumab 40 mg EOW that achieved ASDAS ID (**C**) or ASDAS LDA (**E**) at weeks 12 and 24 from SELECT-PsA 1. Proportions of PsA patients with axial involvement defined by either criterion treated with placebo or upadacitinib 15 mg QD that achieved ASDAS ID (**D**) or ASDAS LDA (**F**) at weeks 12 and 24 from SELECT-PsA 2. ASDAS ID defined as score < 1.3; LDA defined as score < 2.1. ASDAS was analyzed using mixed-effect model for repeated measures and are shown as least squares means with 95% CIs. ASDAS ID and ASDAS LDA were analyzed using Cochran-Mantel–Haenszel tests with non-responder imputation and are shown as response rates with 95% CIs. ****P < 0.0001, ***P < 0.001, **P < 0.01, upadacitinib 15 mg versus placebo; ^##^*P* < 0.01, upadacitinib 15 mg versus adalimumab; nominal *P* values are shown and were not multiplicity controlled. ADA, adalimumab; ASDAS, Ankylosing Spondylitis Disease Activity Score; bDMARD, biologic disease-modifying antirheumatic drug; CI, confidence interval; EOW, every other week; ID, inactive disease; IR, inadequate response; LDA, low disease activity; PBO, placebo; PRO, patient-reported outcome; PsA, psoriatic arthritis; QD, once daily; UPA, upadacitinib

In both studies, greater improvements in ASDAS scores were observed with upadacitinib 15 mg versus placebo at weeks 12 and 24 according to both criteria used to define axial involvement (Fig. 3A, B). A greater improvement in the ASDAS score was also observed with upadacitinib 15 mg versus adalimumab at week 24 in PsA patients with axial involvement defined by investigator judgement alone (Fig. 3A). More PsA patients with axial involvement achieved ASDAS ID with upadacitinib 15 mg versus placebo based on either criterion at weeks 12 and 24 in SELECT PsA 1 (Fig. 3C) and at week 24 in SELECT-PsA 2 (Fig. 3D); results were numerically greater with upadacitinib 15 mg versus placebo at week 12 in SELECT-PsA 2. Greater proportions of PsA patients with axial involvement in both studies achieved ASDAS LDA with upadacitinib 15 mg versus placebo, as well as ASDAS MI (Supplementary Fig. S3A, B) and ASDAS CII (Supplementary Fig. S3C, D), at both timepoints and according to both criteria (Fig. 3E, F). In addition, greater proportions of PsA patients with axial involvement treated with upadacitinib 15 mg versus adalimumab achieved ASDAS MI (investigator alone) or ASDAS CII (both criteria) at week 24 (Supplementary Fig. S3A, C). As stated above for the BASDAI efficacy endpoints, ASDAS and ASDAS responses (ID, LDA, MI, and CII) were similar with upadacitinib 15 mg and adalimumab at week 12 but were numerically higher with upadacitinib 15 mg than adalimumab at week 24, according to both criteria.

Long-term assessment of BASDAI and ASDAS efficacy endpoints in PsA patients with axial involvement treated with upadacitinib 15 mg demonstrated that the clinical improvements observed at weeks 12 and 24 were maintained at week 56 in both studies based on both criteria (Supplementary Table S1, Supplementary Table S2). Furthermore, PsA patients treated with upadacitinib 15 mg showed consistent numerically higher responses than adalimumab across the efficacy endpoints regardless of the criteria used to define axial involvement.

### Safety

In this post-hoc analysis of PsA patients with or without axial involvement from SELECT-PsA 1 (non-bDMARD-IR) and SELECT-PsA 2 (bDMARD-IR), the safety profile of upadacitinib 15 mg though week 56 (Tables 2 and 3, respectively) was generally similar to previously published data from the full study populations [20–22]. Overall, rates of TEAEs were generally similar across sub-groups irrespective of axial involvement status, with only a few differences to note. Compared to patients without axial involvement, patients with axial involvement (defined by either criterion) treated with upadacitinib 15 mg had lower rates of any serious adverse event or any infection, but higher rates of hepatic disorder and anemia in the non-bDMARD-IR population (Table 2), while lower rates of hepatic disorder, but higher rates of opportunistic infection, creatine phosphokinase (CPK) elevation, any malignancy, and malignancy excluding non-melanoma skin cancer (NMSC) in the bDMARD-IR population (Table 3). In SELECT-PsA 1, rates of any serious adverse event and hepatic disorder were higher with adalimumab, while anemia was higher with upadacitinib 15 mg, in patients with axial involvement compared to those without; serious infection and CPK elevation were higher with upadacitinib 15 mg versus adalimumab in patients without axial involvement compared to those with axial involvement (Table 2).

**Table 2:** Summary of Safety Data Through Week 56 From SELECT-PsA 1 (non-bDMARD-IR)

EAER, E/100 PY^a^ (95% CI)	Without Axial Involvement		Investigator Alone		Investigator + PRO-Based	
	UPA 15 mg QD^b^ (*n* = 411; PY = 572.1)	ADA 40 mg EOW (*n* = 302; PY = 448.3)	UPA 15 mg QD^b^ (*n* = 206; PY = 267.0)	ADA 40 mg EOW (*n* = 127; PY = 183.1)	UPA 15 mg QD^b^ (*n* = 158; PY = 206.9)	ADA 40 mg EOW (*n* = 85; PY = 122.9)
Any AE	283.7 (270.1, 297.8)	247.2 (232.8, 262.1)	275.7 (256.1, 296.3)	311.9 (286.8, 338.5)	280.3 (258.0, 304.1)	335.2 (303.6, 369.2)
Any serious AE	10.1 (7.7, 13.1)	8.3 (5.8, 11.4)	6.7 (4.0, 10.7)	12.0 (7.5, 18.2)	7.7 (4.4, 12.6)	15.5 (9.3, 24.1)
Any AE leading to discontinuation of study drug	4.7 (3.1, 6.9)	7.8 (5.4, 10.9)	4.5 (2.3, 7.9)	6.6 (3.4, 11.4)	4.3 (2.0, 8.3)	8.1 (3.9, 15.0)
All deaths (n/100 PY)^c^	0.3 (0.0, 1.3)	0	0	0.5 (0.0, 3.0)	0	0.8 (0.0, 4.5)
AEs of special interest						
Any infection	100.3 (92.3, 108.9)	70.5 (62.9, 78.7)	83.1 (72.6, 94.8)	72.6 (60.8, 86.1)	80.7 (68.9, 93.9)	73.2 (58.9, 90.0)
Serious infection	3.1 (1.9, 5.0)	0.9 (0.2, 2.3)	2.2 (0.8, 4.9)	2.2 (0.6, 5.6)	2.4 (0.8, 5.6)	2.4 (0.5, 7.1)
Opportunistic infection^d^	0.3 (0.0, 1.3)	0	0.4 (0.0, 2.1)	0	0.5 (0.0, 2.7)	0
Herpes zoster	3.7 (2.3, 5.6)	0.7 (0.1, 2.0)	4.5 (2.3, 7.9)	0	4.8 (2.3, 8.9)	0
Active tuberculosis	0	0	0	0	0	0
GI perforation (adjudicated)	0.3 (0.0, 1.3)	0	0	0	0	0
Hepatic disorder	16.6 (13.4, 20.3)	19.0 (15.1, 23.4)	24.3 (18.8, 31.0)	39.3 (30.8, 49.5)	27.5 (20.9, 35.7)	45.6 (34.4, 59.2)
Anemia	1.9 (1.0, 3.4)	1.3 (0.5, 2.9)	5.2 (2.9, 8.8)	2.2 (0.6, 5.6)	4.3 (2.0, 8.3)	1.6 (0.2, 5.9)
Neutropenia	2.3 (1.2, 3.9)	3.8 (2.2, 6.1)	2.6 (1.1, 5.4)	5.5 (2.6, 10.0)	2.9 (1.1, 6.3)	4.9 (1.8, 10.6)
Lymphopenia	2.6 (1.5, 4.3)	0.2 (0.0, 1.2)	4.5 (2.3, 7.9)	0	2.4 (0.8, 5.6)	0
CPK elevation	12.8 (10.0, 16.0)	5.8 (3.8, 8.5)	10.1 (6.7, 14.7)	10.9 (6.7, 16.9)	10.1 (6.3, 15.5)	11.4 (6.2, 19.1)
Renal dysfunction	0	0	0.7 (0.1, 2.7)	0	0.5 (0.0, 2.7)	0
Any malignancy	1.1 (0.4, 2.3)	0.7 (0.1, 2.0)	1.1 (0.2, 3.3)	1.6 (0.3, 4.8)	1.5 (0.3, 4.3)	0.8 (0.0, 4.5)
Malignancy (excl. NMSC)	0.5 (0.1, 1.5)	0.7 (0.1, 2.0)	0.7 (0.1, 2.7)	0.5 (0.0, 3.0)	1.0 (0.1, 3.5)	0.8 (0.0, 4.5)
NMSC	0.7 (0.2, 1.8)	0	0.4 (0.0, 2.1)	1.1 (0.1, 4.0)	0.5 (0.0, 2.7)	0
Lymphoma	0	0	0	0	0	0
MACE (adjudicated)^e^	0.5 (0.1, 1.5)	0.2 (0.0, 1.2)	0	1.1 (0.1, 4.0)	0	1.6 (0.2, 5.9)
VTE (adjudicated)^f^	0.3 (0.0, 1.3)	0.2 (0.0, 1.2)	0.4 (0.0, 2.1)	0.6 (0.0, 3.1)	0.5 (0.0, 2.7)	0.8 (0.0, 4.6)

*ADA* Adalimumab, *AE* Adverse event, *bDMARD* Biologic disease-modifying antirheumatic drug, *CI* Confidence interval, *CPK* Creatine phosphokinase, *E* Event, *EAER* Exposure-adjusted event rate, *EOW* Every other week, *IR* Inadequate response, *MACE* Major adverse cardiovascular event, *NMSC* Non-melanoma skin cancer, *PsA* Psoriatic arthritis, *PY* Patient-years, *QD* Once daily, *UPA* Upadacitinib, *VTE* Venous thromboembolism

^a^Safety data presented as events per 100 patient-years (with 95% CIs), unless indicated

^b^Patients in the UPA 15 mg QD group include those who were assigned to UPA 15 mg QD at baseline, as well as those switched from placebo to UPA 15 mg QD at week 24

^c^Two deaths were reported with upadacitinib 15 mg from the sub-group without axial involvement, one death with adalimumab from the investigator alone sub-group, and one death with adalimumab from the investigator judgement and PRO-based sub-group

^d^Opportunistic infections excluding tuberculosis and herpes zoster

^e^Major adverse cardiovascular events defined as non-fatal myocardial infarction, non-fatal stroke, and cardiovascular death

^f^Venous thromboembolism includes deep vein thrombosis (DVT) and pulmonary embolism (PE) (fatal and non-fatal)

**Table 3:** Summary of Safety Data Through Week 56 From SELECT-PsA 2 (bDMARD-IR)

EAER, E/100 PY^a^ (95% CI)	Without Axial Involvement	Investigator Alone	Investigator + PRO-Based
	UPA 15 mg QD^b^ (*n* = 184; PY = 272.5)	UPA 15 mg QD^b^ (*n* = 106; PY = 146.9)	UPA 15 mg QD^b^ (*n* = 81; PY = 113.4)
Any AE	259.8 (241.0, 279.7)	262.1 (236.6, 289.6)	254.0 (225.5, 285.1)
Any serious AE	12.5 (8.6, 17.4)	17.7 (11.6, 25.9)	18.5 (11.5, 28.3)
Any AE leading to discontinuation of study drug	11.0 (7.4, 15.7)	8.2 (4.2, 14.3)	7.9 (3.6, 15.1)
All deaths (n/100 PY)	0	0	0
AEs of special interest			
Any infection	91.0 (80.0, 103.1)	87.1 (72.7, 103.6)	82.0 (66.2, 100.5)
Serious infection	2.9 (1.3, 5.8)	2.0 (0.4, 6.0)	1.8 (0.2, 6.4)
Opportunistic infection^c^	0.4 (0.0, 2.0)	1.4 (0.2, 4.9)	1.8 (0.2, 6.4)
Herpes zoster	4.0 (2.0, 7.2)	3.4 (1.1, 7.9)	3.5 (1.0, 9.0)
Active tuberculosis	0	0	0
GI perforation (adjudicated)	0	0	0
Hepatic disorder	5.9 (3.4, 9.5)	2.7 (0.7, 7.0)	2.6 (0.5, 7.7)
Anemia	2.6 (1.0, 5.3)	1.4 (0.2, 4.9)	1.8 (0.2, 6.4)
Neutropenia	1.5 (0.4, 3.8)	0	0
Lymphopenia	1.1 (0.2, 3.2)	0	0
CPK elevation	4.4 (2.3, 7.7)	6.8 (3.3, 12.5)	7.9 (3.6, 15.1)
Renal dysfunction	0.7 (0.1, 2.7)	0	0
Any malignancy	1.5 (0.4, 3.8)	4.2 (1.5, 9.1)	5.4 (2.0, 11.8)
Malignancy (excl. NMSC)	0.4 (0.0, 2.0)	2.7 (0.7, 7.0)	3.5 (1.0, 9.0)
NMSC	1.1 (0.2, 3.2)	1.4 (0.2, 5.0)	1.8 (0.2, 6.5)
Lymphoma	0.7 (0.1, 2.7)	0	0
MACE (adjudicated)^d^	0	0.7 (0.0, 3.8)	0.9 (0.0, 5.0)
VTE (adjudicated)^e^	0.4 (0.0, 2.0)	0	0

*AE* Adverse event, *bDMARD* Biologic disease-modifying antirheumatic drug, *CI* Confidence interval, *CPK* Creatine phosphokinase, *E* Event, *EAER* Exposure-adjusted event rate, *EOW* Every other week, *IR* Inadequate response, *MACE* Major adverse cardiovascular event, *NMSC* Non-melanoma skin cancer, *PsA* Psoriatic arthritis, *PY* Patient-years, *QD* Once daily, *UPA* Upadacitinib, *VTE* Venous thromboembolism

^a^Safety data presented as events per 100 patient-years (with 95% CIs), unless indicated

^b^Patients in the UPA 15 mg QD group include those who were assigned to UPA 15 mg QD at baseline, as well as those switched from placebo to UPA 15 mg QD at week 24

^c^Opportunistic infections excluding tuberculosis and herpes zoster

^d^Major adverse cardiovascular events defined as non-fatal myocardial infarction, non-fatal stroke, and cardiovascular death

^e^Venous thromboembolism includes deep vein thrombosis (DVT) and pulmonary embolism (PE) (fatal and non-fatal)

## Discussion

Here, we demonstrate that based on two different definitions of axial involvement, patients with active PsA and axial involvement had greater clinical responses for measures related to their axial symptoms (as determined by BASDAI and ASDAS) with upadacitinib 15 mg (approved dose) versus placebo at week 12, which were maintained or further improved at week 24 in both SELECT-PsA 1 (non-bDMARD-IR population) and SELECT-PsA 2 (bDMARD-IR population). In addition, SELECT-PsA 1 patients often had similar or greater numerical responses compared to adalimumab across multiple endpoints related to axial disease activity. Clinical improvements observed with upadacitinib 15 mg were maintained long-term at week 56. Responses were consistent across patient populations (non-bDMARD-IR and bDMARD-IR), endpoints (BASDAI, individual BASDAI components including question 2 related to back pain, and ASDAS), and assessments for axial involvement (investigator alone or investigator and PRO-based criteria), demonstrating support for inhibition of the JAK-STAT signaling pathway to improve axial symptoms in patients with active PsA. Complementary to the efficacy of upadacitinib in PsA with axial involvement, upadacitinib 15 mg reduced disease activity and axial inflammation (as detected by magnetic resonance imaging [MRI]), as well as improved function, in patients with AS or non-radiographic axial spondyloarthritis (nr-axSpA), providing further support from dedicated AS and axSpA studies for the efficacy of JAK inhibitors to treat axial disease [16, 17, 27, 28].

PsA with axial involvement remains a topic of high clinical interest due to limited data on this specific patient population and no agreed upon classification or diagnostic criteria for axial involvement. Previous studies have utilized a variety of criteria to classify axial involvement in PsA, including applying CASPAR or Assessment of SpondyloArthritis international Society (ASAS) criteria, BASDAI cut-offs, presence of inflammatory back pain, sacroiliitis on imaging, and/or other criteria [1, 25, 29]. Given the lack of clear criteria, the presence of axial involvement (‘psoriatic spondylitis’) was determined by the investigators in the SELECT-PsA trials based on their judgement of the totality of clinical information that was available to them. We further strengthened the recognition of inflammatory axial involvement by applying PRO-based criteria (BASDAI score ≥ 4 and BASDAI question 2 score [AS neck, back, or hip pain] ≥ 4 at baseline) to confirm active axial disease in patients from the SELECT-PsA trials [25]. This analysis shows that PsA patients with axial involvement treated with upadacitinib 15 mg using either of the predefined criteria showed improvements in measures of axial disease, including ASDAS and modified BASDAI (excluding question 3 related to peripheral joint pain).

In this post-hoc analysis of the SELECT-PsA trials, the safety profile of upadacitinib 15 mg was generally similar between patients with axial involvement using either criterion, as well as those without axial involvement. Minor variability in the rates of some TEAEs (eg, hepatic disorder in SELECT-PsA 1 [non-bDMARD-IR] or CPK elevation in SELECT-PsA 2 [bDMARD-IR]) were noted in patients with axial involvement, but no clear patterns were observed across the two studies. No new safety risks were identified with upadacitinib 15 mg treatment in this post-hoc analysis of the SELECT-PsA studies [20, 21, 30, 31].

Potential limitations of this post-hoc analysis should also be considered. First, axial involvement in the SELECT-PsA trials was determined by investigator judgement and was not consistently confirmed by imaging in all patients. Improvements in active MRI inflammation in the spine and sacroiliac joints has been demonstrated previously with upadacitinib treatment in patients with AS or nr-axSpA [16, 17, 27, 28]. In addition, similar to our analysis, the secukinumab MAXIMISE trial did not require imaging confirmation, but instead identified patients with PsA and axial manifestations based on CASPAR criteria, active spinal disease with a BASDAI score ≥ 4, and a spinal pain Visual Analogue Score ≥ 40/100 at baseline, with improvements in MRI scores observed (in patients with available imaging) in the spine and sacroiliac joints following treatment [25]. Although the lack of imaging is a limitation of the SELECT-PsA trials, the diagnosis of axial involvement by treating physicians based on available clinical information does reflect real-world clinical practice, where imaging may not always be routinely conducted to confirm a diagnosis. In addition, HLA-B27 status, which has been identified more often in patients with axial involvement [1] and has been associated with greater disease severity in PsA, was not assessed in either SELECT-PsA trial. Furthermore, as is common to post-hoc analyses, statistical comparisons for the BASDAI and ASDAS efficacy endpoints were not multiplicity controlled, and only nominal *P* values have been displayed in this manuscript.

## Conclusions

In summary, irrespective of the predefined assessment for axial involvement applied (investigator judgement alone or both investigator judgement and PRO-based criteria), patients with active PsA and axial involvement demonstrated improvements in their axial symptoms with upadacitinib 15 mg, often with greater numerical responses versus adalimumab, which were maintained over long-term follow-up (56 weeks) in two phase 3 studies. Safety results for upadacitinib 15 mg were generally comparable between patients with or without axial involvement. These data provide important information for treating clinicians on the efficacy and safety of upadacitinib 15 mg in axial disease and may help guide treatment decisions for PsA patients with axial involvement.

## Supplementary Information

**Additional file 1:** **Supplementary Fig. S1. **BASDAI Components at Weeks 12 and 24 From SELECT-PsA 1(non-bDMARD-IR). **Supplementary Fig. S2. **BASDAI Components at Weeks 12 and 24 From SELECT-PsA 2 (bDMARD-IR). **Supplementary Fig. S3. **ASDAS Major Improvement (MI) and ASDAS Clinically Important Improvement (CII) at Weeks 12 and 24 From SELECT-PsA 1 (non-bDMARD-IR) and SELECT-PsA 2 (bDMARD-IR). **Supplementary Table S1. **Efficacy Endpoints at Week 56 From SELECT-PsA 1 (non-bDMARD-IR). **Supplementary Table S2. **Efficacy Endpoints at Week 56 From SELECT-PsA 2 (bDMARD-IR). 

## Data Availability

AbbVie is committed to responsible data sharing regarding the clinical trials we sponsor. This includes access to anonymized, individual, and trial-level data (analysis data sets), as well as other information (eg, protocols, clinical study reports, or analysis plans), as long as the trials are not part of an ongoing or planned regulatory submission. This includes requests for clinical trial data for unlicensed products and indications. These clinical trial data can be requested by any qualified researchers who engage in rigorous, independent scientific research, and will be provided following review and approval of a research proposal, Statistical Analysis Plan (SAP), and execution of a Data Sharing Agreement (DSA). Data requests can be submitted at any time after approval in the US and Europe and after acceptance of this manuscript for publication. The data will be accessible for 12 months, with possible extensions considered. For more information on the process or to submit a request, visit the following link: https://www.abbvieclinicaltrials.com/hcp/data-sharing/.html.

## Abbreviations

ACR: American College of Rheumatology
ADA: Adalimumab
AS: Ankylosing spondylitis
ASADAS: Ankylosing Spondylitis Disease Activity Score
ASAS: Assessment of SpondyloArthritis international Society
axSpA: Axial spondyloarthritis
BASDAI: Bath Ankylosing Spondylitis Disease Activity Index
BASDAI50: ≥ 50% Improvement from baseline in BASDAI
bDMARD: Biologic disease-modifying antirheumatic drug
BSA: Body surface area
CASPAR: Classification Criteria for Psoriatic Arthritis
CI: Confidence interval
CII: Clinically important improvement
CPK: Creatine phosphokinase
CRP: C-reactive protein
CTCAE: Common Terminology Criteria for Adverse Events
DSA: Data Sharing Agreement
DVT: Deep vein thrombosis
EAER: Exposure-adjusted event rates
EOW: Every other week
GCP: Good Clinical Practice
GI: Gastrointestinal
hsCRP: High-sensitivity CRP
ICH: International Council for Harmonisation of Technical Requirements for Pharmaceuticals for Human Use
ID: Inactive disease
IEC: Independent ethics committee
IR: Inadequate response
IRB: Institutional review board
JAK: Janus kinase
LDA: Low disease activity
MACE: Major adverse cardiovascular event
MedDRA: Medical Dictionary for Regulatory Activities
MI: Major improvement
MMRM: Mixed-effect model for repeated measures
MRI: Magnetic resonance imaging
NMSC: Non-melanoma skin cancer
nr-axSpA: Non-radiographic axial spondyloarthritis
NRI: Non-responder imputation
PBO: Placebo
PE: Pulmonary embolism
PRO: Patient-reported outcome
PsA: Psoriatic arthritis
PY: Patient-year(s)
QD: Once daily
SAP: Statistical Analysis Plan
SJC66: Swollen joint count 66
STAT: Signal transducer and activator of transcription
TEAE: Treatment-emergent adverse event
TJC68: Tender joint count 68
UPA: Upadacitinib
UPA15: Upadacitinib 15 mg
VTE: Venous thromboembolism
