# Supplementary material for: Efficacy and safety of upadacitinib in patients with active psoriatic arthritis and axial involvement: results from two phase 3 studies
Source: Arthritis Res Ther. 2023 Apr 10;25:56. doi: 10.1186/s13075-023-03027-5 (PMC10084601; doi:10.1186/s13075-023-03027-5)
Supplement: Supplementary file 1 — Additional file 1: Supplementary Fig. S1. BASDAI Components at Weeks 12 and 24 From SELECT-PsA 1(non-bDMARD-IR). Supplementary Fig. S2. BASDAI Components at Weeks 12 and 24 From SELECT-PsA 2 (bDMARD-IR). Supplementary Fig. S3. ASDAS Major Improvement (MI) and ASDAS Clinically Important Improvement (CII) at Weeks 12 and 24 From SELECT-PsA 1 (non-bDMARD-IR) and SELECT-PsA 2 (bDMARD-IR). Supplementary Table S1. Efficacy Endpoints at Week 56 From SELECT-PsA 1 (non-bDMARD-IR). Supplementary Table S2. Efficacy Endpoints at Week 56 From SELECT-PsA 2 (bDMARD-IR). [file 13075_2023_3027_MOESM1_ESM.pdf]

## SUPPLEMENTARY MATERIAL

**Efficacy and Safety of Upadacitinib in Patients With Active Psoriatic Arthritis and Axial Involvement: Results From Two Phase 3 Studies**

Xenofon Baraliakos, MD, PhD; Roberto Ranza, MD; Andrew Östör, MD; Francesco Ciccia, MD, PhD; Laura C. Coates, MBChB, MRCP, PhD; Simona Rednic, MD, PhD; Jessica Walsh, MD[7]; Kevin Douglas, MD; Tianming Gao, PhD; Koji Kato, MBBS; In-Ho Song, MD; Fabiana Ganz, PhD, MPH; Atul Deodhar, MD, MRCP

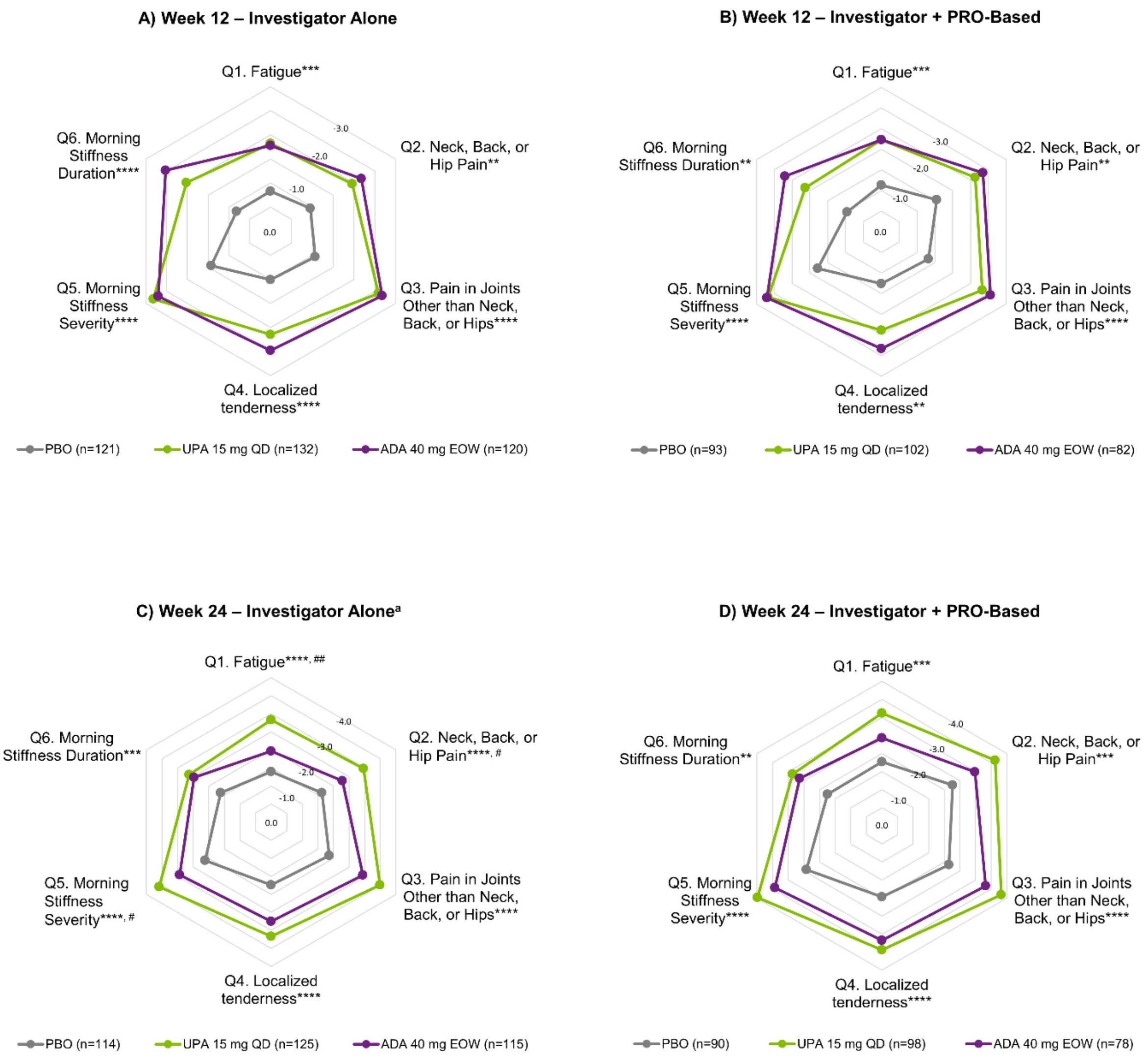


**Supplementary Fig. S1. BASDAI Components at Weeks 12 and 24 From SELECT-PsA 1 (non-bDMARD-IR)**

Mean change from baseline in individual BASDAI components for PsA patients with axial involvement defined by investigator judgement alone at week 12 (A) or week 24 (C), as well as investigator judgement and PRO-based criteria at week 12 (B) or week 24 (D), treated with placebo, upadacitinib 15 mg QD, or adalimumab 40 mg EOW from SELECT-PsA 1. BASDAI question 1 (Q1): How would you describe the overall level of fatigue/tiredness you have experienced? BASDAI question 2 (Q2): How would you describe the overall level of AS (ankylosing spondylitis) neck, back, or hip pain you have had? BASDAI

question 3 (Q3): How would you describe the overall level of pain/swelling in joints other than neck, back, or hips you have had? BASDAI question 4 (Q4): How would you describe the overall level of discomfort you have had from any areas tender to touch or pressure? BASDAI question 5 (Q5): How would you describe the overall level of discomfort you have had from the time you wake up? BASDAI question 6 (Q6): How long does your morning stiffness last from the time you wake up? [a]For BASDAI questions 2 and 3, n=115 for placebo and n=114 for adalimumab for the investigator alone sub-group at week 24. Data were analyzed using mixed-effect model for repeated measures and are shown as least squares means. ****$P<0.0001$, ***$P<0.001$, **$P<0.01$, *$P<0.05$, upadacitinib 15 mg versus placebo; $^{\#\#}P<0.01$, $^{\#}P<0.05$, upadacitinib 15 mg versus adalimumab; nominal *P* values are shown and were not multiplicity controlled. ADA, adalimumab; BASDAI, Bath Ankylosing Spondylitis Disease Activity Index; bDMARD, biologic disease-modifying antirheumatic drug; EOW, every other week; IR, inadequate response; PBO, placebo; PRO, patient-reported outcome; PsA, psoriatic arthritis; QD, once daily; UPA, upadacitinib.

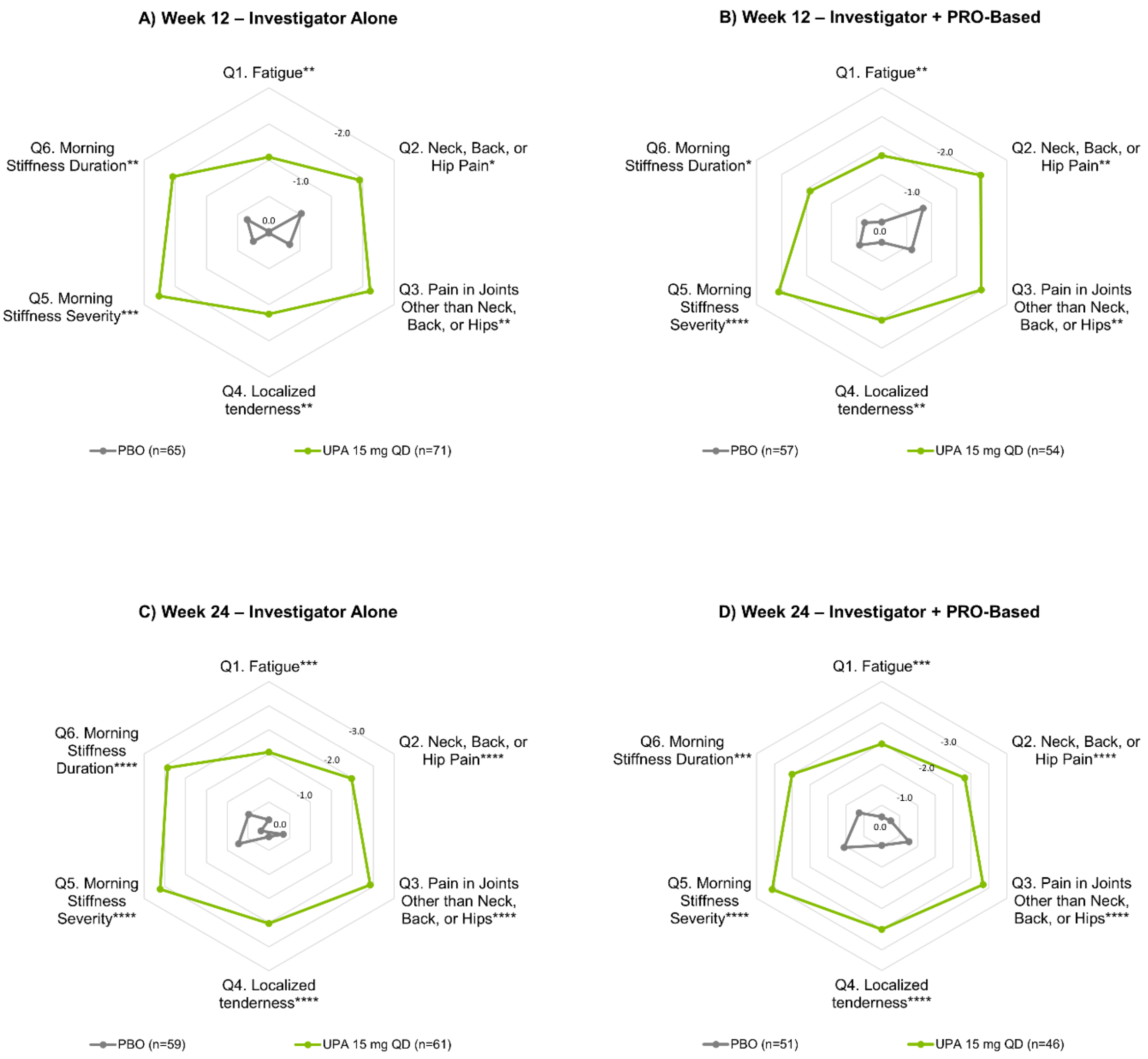


**Supplementary Fig. S2. BASDAI Components at Weeks 12 and 24 From SELECT-PsA 2 (bDMARD-IR)**

Mean change from baseline in individual BASDAI components for PsA patients with axial involvement defined by investigator judgement alone at week 12 (A) or week 24 (C), as well as investigator judgement and PRO-based criteria at week 12 (B) or week 24 (D), treated with placebo or upadacitinib 15 mg QD from SELECT-PsA 2. BASDAI question 1 (Q1): How would you describe the overall level of fatigue/tiredness you have experienced? BASDAI question 2 (Q2): How would you describe the overall level of AS (ankylosing spondylitis) neck, back, or hip pain you have had? BASDAI question 3 (Q3): How

would you describe the overall level of pain/swelling in joints other than neck, back, or hips you have had? BASDAI question 4 (Q4): How would you describe the overall level of discomfort you have had from any areas tender to touch or pressure? BASDAI question 5 (Q5): How would you describe the overall level of discomfort you have had from the time you wake up? BASDAI question 6 (Q6): How long does your morning stiffness last from the time you wake up? Data were analyzed using mixed-effect model for repeated measures and are shown as least squares means. ****$P < 0.0001$, ***$P < 0.001$, **$P < 0.01$, *$P < 0.05$, upadacitinib 15 mg versus placebo; nominal *P* values are shown and were not multiplicity controlled. BASDAI, Bath Ankylosing Spondylitis Disease Activity Index; bDMARD, biologic disease-modifying antirheumatic drug; IR, inadequate response; PBO, placebo; PRO, patient-reported outcome; PsA, psoriatic arthritis; QD, once daily; UPA, upadacitinib.

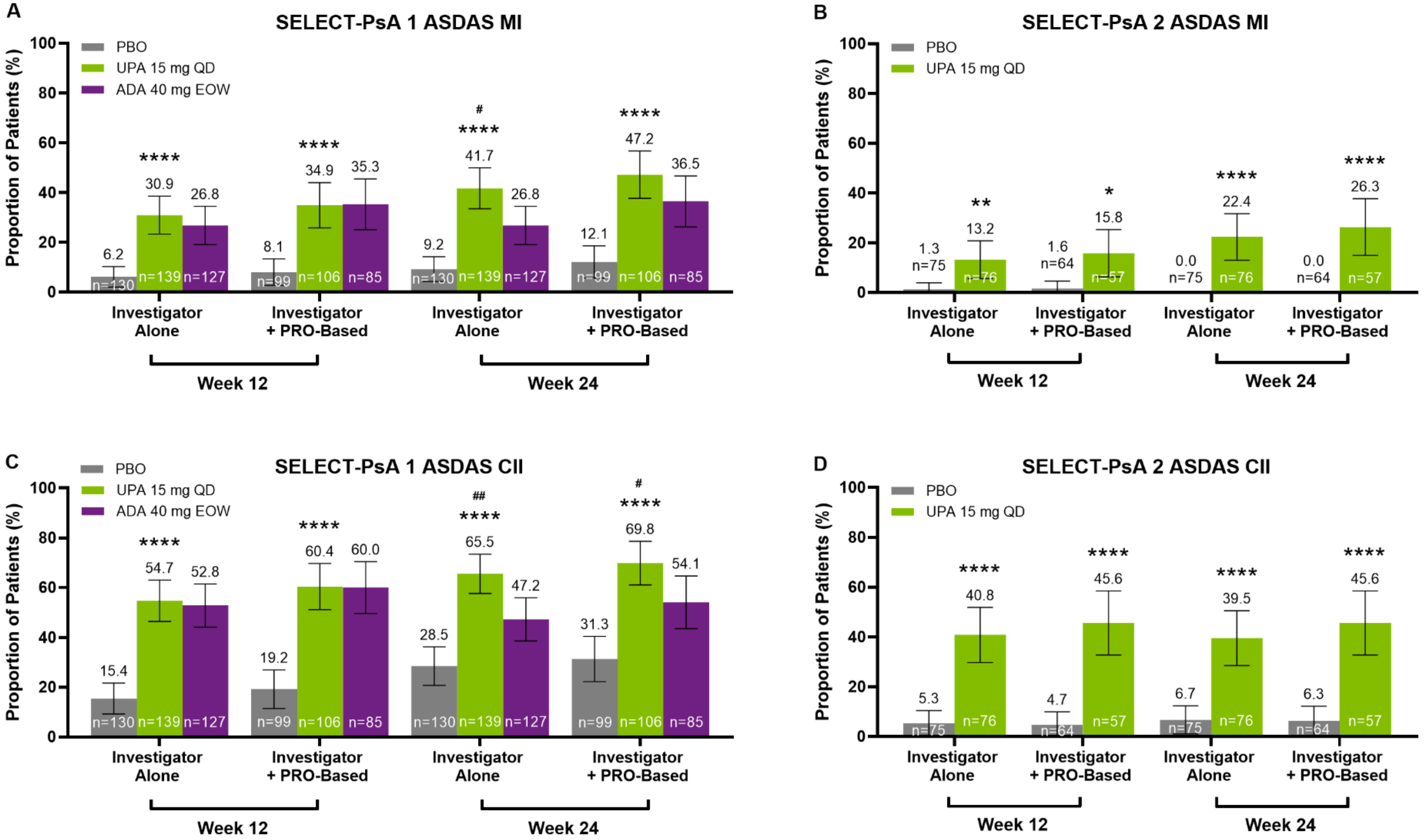


**Supplementary Fig. S3. ASDAS Major Improvement (MI) and ASDAS Clinically Important Improvement (CII) at Weeks 12 and 24 From SELECT-PsA 1 (non-bDMARD-IR) and SELECT-PsA 2 (bDMARD-IR)**

Proportions of PsA patients with axial involvement defined by investigator judgement alone or investigator judgement and PRO-based criteria treated with placebo, upadacitinib 15 mg QD, or adalimumab 40 mg EOW that achieved ASDAS MI (A) or ASDAS CII (C) at weeks 12 and 24 from SELECT-PsA 1. Proportions of PsA patients with axial involvement defined by either criterion treated with placebo or upadacitinib 15 mg QD that achieved ASDAS MI (B) or ASDAS CII (D) at weeks 12 and 24 from SELECT-PsA 2. ASDAS MI defined as ≥ 2.0 decrease from baseline; CII defined as ≥ 1.1 decrease from baseline. Data were analyzed using Cochran-Mantel-Haenszel tests with non-responder imputation and are shown as response rates with 95% CIs. ****$P < 0.0001$, **$P < 0.01$, *$P < 0.05$, upadacitinib 15 mg versus placebo; ##$P < 0.01$, #$P < 0.05$, upadacitinib 15 mg versus adalimumab; nominal *P* values are shown and were not multiplicity controlled. ADA, adalimumab; ASDAS, Ankylosing Spondylitis Disease Activity Score; bDMARD, biologic disease-modifying antirheumatic drug; CI, confidence interval; CII, clinically important improvement; EOW, every other week; IR, inadequate response; MI, major improvement; PBO, placebo; PRO, patient-reported outcome; PsA, psoriatic arthritis; QD, once daily; UPA, upadacitinib.

## Supplementary Table S1. Efficacy Endpoints at Week 56 From SELECT-PsA 1 (non-bDMARD-IR)

| | **Investigator Alone** | | | **Investigator + PRO-Based** | | |
|---|---|---|---|---|---|---|
| | PBO to UPA 15 mg QD[a] | UPA 15 mg QD | ADA 40 mg EOW | PBO to UPA 15 mg QD[a] | UPA 15 mg QD | ADA 40 mg EOW |
| Mean change from baseline (MMRM):[b] | n = 63[c] | n = 116[c] | n = 107[c] | n = 48[c] | n = 89[c] | n = 74[c] |
| Overall BASDAI | -3.06 (-3.57, -2.55) | -3.27 (-3.66, -2.89) | -2.84 (-3.23, -2.44) | -3.31 (-3.92, -2.69) | -3.78 (-4.25, -3.31) | -3.25 (-3.75, -2.74) |
| Modified BASDAI (excl. question 3)[d] | -2.90 (-3.41, -2.40) | -3.16 (-3.54, -2.77) | -2.74 (-3.14, -2.35) | -3.18 (-3.79, -2.57) | -3.68 (-4.14, -3.21) | -3.17 (-3.67, -2.67) |
| BASDAI question 2[e] | -2.53 (-3.17, -1.89) | -3.06 (-3.54, -2.57) | -2.49 (-2.99, -1.98) | -3.15 (-3.92, -2.38) | -3.91 (-4.50, -3.33) | -3.20 (-3.83, -2.57) |
| ASDAS (CRP) | -1.68 (-1.93, -1.44) | -1.77 (-1.96, -1.58) | -1.59 (-1.79, -1.40) | -1.77 (-2.06, -1.48) | -1.94 (-2.16, -1.72) | -1.76 (-1.99, -1.52) |
| Proportion of patients (NRI): [f] | n = 73 | n = 139 | n = 127 | n = 56 | n = 106 | n = 85 |
| BASDAI50 | 45.2 (33.8, 56.6) | 58.3 (50.1, 66.5) | 46.5 (37.8, 55.1) | 41.1 (28.2, 54.0) | 60.4 (51.1, 69.7) | 49.4 (38.8, 60.0) |
| ASDAS ID[g] | 34.2 (23.4, 45.1) | 41.7 (33.5, 49.9) | 37.8 (29.4, 46.2) | 26.8 (15.2, 38.4) | 39.6 (30.3, 48.9) | 34.1 (24.0, 44.2) |
| ASDAS LDA[g] | 56.2 (44.8, 67.5) | 59.7 (51.6, 67.9) | 52.8 (44.1, 61.4) | 51.8 (38.7, 64.9) | 59.4 (50.1, 68.8) | 49.4 (38.8, 60.0) |
| ASDAS MI[g] | 30.1 (19.6, 40.7) | 41.7 (33.5, 49.9)[#] | 27.6 (19.8, 35.3) | 32.1 (19.9, 44.4) | 46.2 (36.7, 55.7) | 36.5 (26.2, 46.7) |
| ASDAS CII[g] | 61.6 (50.5, 72.8) | 60.4 (52.3, 68.6) | 49.6 (40.9, 58.3) | 60.7 (47.9, 73.5) | 63.2 (54.0, 72.4) | 57.6 (47.1, 68.2) |

ADA, adalimumab; ASDAS, Ankylosing Spondylitis Disease Activity Score; BASDAI, Bath Ankylosing Spondylitis Disease Activity Index; bDMARD, biologic disease-modifying antirheumatic drug; BASDAI50, ≥ 50% improvement from baseline in BASDAI; CI, confidence interval; CII, clinically important improvement; EOW, every other week; ID, inactive disease; IR, inadequate response; LDA, low disease activity; MI, major improvement; MMRM, mixed-effect model for repeated measures; NRI, non-responder imputation; PBO, placebo; PRO, patient-reported outcome; PsA, psoriatic arthritis; QD, once daily; UPA, upadacitinib.

[a]Patients randomized to PBO at baseline were switched to blinded UPA 15 mg QD at week 24 of the trial.

[b]MMRM used for continuous endpoints; data shown as LS means with 95% CIs.

[c]Sample sizes for ASDAS varied from that shown in the table: Investigator alone (PBO to UPA 15 mg QD, n = 62; UPA 15 mg QD, n = 113; ADA 40 mg EOW, n = 102) and Investigator + PRO-Based (PBO to UPA 15 mg QD, n = 47; UPA 15 mg QD, n = 87; ADA 40 mg EOW, n = 71).

[d]Modified BASDAI, excluding question 3 - How would you describe the overall level of pain/swelling in joints other than neck, back, or hips you have had?

[e]BASDAI question 2 - How would you describe the overall level of AS (ankylosing spondylitis) neck, back, or hip pain you have had?

[f]Cochran-Mantel-Haenszel tests with NRI used for binary endpoints; data shown as response rate with 95% CIs.

[g]ASDAS ID was defined as score < 1.3; LDA was defined as score < 2.1; MI was defined as ≥ 2.0 decrease from baseline; CII was defined as ≥ 1.1 decrease from baseline.

[#]$P < 0.05$, upadacitinib 15 mg versus adalimumab; nominal *P* values are shown and were not multiplicity controlled.

## Supplementary Table S2. Efficacy Endpoints at Week 56 From SELECT-PsA 2 (bDMARD-IR)

| | Investigator Alone | | Investigator + PRO-Based | |
|---|---|---|---|---|
| | PBO to UPA 15 mg QD[a] | UPA 15 mg QD | PBO to UPA 15 mg QD[a] | UPA 15 mg QD |
| Mean change from baseline (MMRM):[b] | n = 24 | n = 54[c] | n = 19 | n = 42[c] |
| Overall BASDAI | -2.28 (-3.07, -1.49) | -2.15 (-2.68, -1.61) | -2.88 (-3.72, -2.04) | -2.63 (-3.21, -2.04) |
| Modified BASDAI (excl. question 3)[d] | -2.20 (-2.98, -1.42) | -2.11 (-2.64, -1.58) | -2.82 (-3.66, -1.98) | -2.61 (-3.20, -2.02) |
| BASDAI question 2[e] | -2.02 (-3.03, -1.00) | -2.03 (-2.72, -1.34) | -2.73 (-3.90, -1.55) | -2.58 (-3.40, -1.76) |
| ASDAS (CRP) | -1.13 (-1.49, -0.77) | -1.32 (-1.56, -1.07) | -1.26 (-1.65, -0.86) | -1.47 (-1.75, -1.19) |
| Proportion of patients (NRI):[f] | n = 40 | n = 76 | n = 33 | n = 57 |
| BASDAI50 | 25.0 (11.6, 38.4) | 31.6 (21.1, 42.0) | 24.2 (9.6, 38.9) | 31.6 (19.5, 43.6) |
| ASDAS ID[g] | 20.0 (7.6, 32.4) | 25.0 (15.3, 34.7) | 18.2 (5.0, 31.3) | 19.3 (9.1, 29.5) |
| ASDAS LDA[g] | 32.5 (18.0, 47.0) | 43.4 (32.3, 54.6) | 27.3 (12.1, 42.5) | 43.9 (31.0, 56.7) |
| ASDAS MI[g] | 15.0 (3.9, 26.1) | 21.1 (11.9, 30.2) | 18.2 (5.0, 31.3) | 24.6 (13.4, 35.7) |
| ASDAS CII[g] | 27.5 (13.7, 41.3) | 35.5 (24.8, 46.3) | 27.3 (12.1, 42.5) | 38.6 (26.0, 51.2) |

ASDAS, Ankylosing Spondylitis Disease Activity Score; BASDAI, Bath Ankylosing Spondylitis Disease Activity Index; bDMARD, biologic disease-modifying antirheumatic drug; BASDAI50, ≥ 50% improvement from baseline in BASDAI; CI, confidence interval; CII, clinically important improvement; EOW, every other week; ID, inactive disease; IR, inadequate response; LDA, low disease activity; MI, major improvement; MMRM, mixed-effect model for repeated measures; NRI, non-responder imputation; PBO, placebo; PRO, patient-reported outcome; PsA, psoriatic arthritis; QD, once daily; UPA, upadacitinib.

[a]Patients randomized to PBO at baseline were switched to blinded UPA 15 mg QD at week 24 of the trial.

[b]MMRM used for continuous endpoints; data shown as LS means with 95% CIs.

[c]Sample sizes for ASDAS varied from that shown in the table: Investigator alone (UPA 15 mg QD, n = 52) and Investigator + PRO-Based (UPA 15 mg QD, n = 41).

[d]Modified BASDAI, excluding question 3 - How would you describe the overall level of pain/swelling in joints other than neck, back, or hips you have had?

[e]BASDAI question 2 - How would you describe the overall level of AS (ankylosing spondylitis) neck, back, or hip pain you have had?

[f]Cochran-Mantel-Haenszel tests with NRI used for binary endpoints; data shown as response rate with 95% CIs.

[g]ASDAS ID was defined as score < 1.3; LDA was defined as score < 2.1; MI was defined as ≥ 2.0 decrease from baseline; CII was defined as ≥ 1.1 decrease from baseline.
